# Supplementary material for: Patterns of Intron Gain and Loss in Fungi
Source: PLoS Biol. 2004 Nov 30;2(12):e422. doi: 10.1371/journal.pbio.0020422 (PMC532390; doi:10.1371/journal.pbio.0020422)
Supplement: Table S1 — Also available at http://genes.mit.edu/NielsenEtAl/. (4.3 MB ZIP). [file pbio.0020422.st001.zip › NielsenEtAl/html/1429.html]

AN8498.1.NCU00825.1.MG05855.1.FG06725.1


```
 CLUSTAL W (1.82) Multiple Sequence Alignments - Introns Inserted


Sequence 1: MG05855.1	627 aa
Sequence 2: FG06725.1	570 aa
Sequence 3: NCU00825.1	650 aa
Sequence 4: AN8498.1	678 aa
Alignment Length: 736 aa
Number Identitical Residues: 118 aa
Alignment Score (without introns) 7395


MG05855.1 	MSVAKFTQHLGTAPEGAP~VGSETSKPKSRRVKAYWAGIGLALLWWVGVKQTHFILSDAT
NCU00825.1	---MGRTQGAGHRVSAIT1VALAILVVLSLS----------DLSALSWTARPNWSTTNTV
FG06725.1 	-------MNEKTTSEAMP~LLRDRLQQRPR-------------------------KSNVS
AN8498.1  	--MEDGKHHSSSRQDGEY~IWPRGRTRRSGSPTLLALTVLAGLSWWLFPISYWDIEAKAS
          	      .       ..   :        .   .                       :.. 

MG05855.1 	AKVNPDDSSTSRQQHNMSFQISWAD0IQPSTT-LRWQECYTGQYDCARLD0VPMDWQNPS
NCU00825.1	YPLNKDQKKEDK-------EFNWAD~ITPGPSPIQWHPCYDDELDCARLN~LPMDWQQPE
FG06725.1 	TVLG-----------------LLAA0IKPRRD-LRWESCYD-KFECARLD0VPMDWLNPS
AN8498.1  	ASPASEN------------QFSWTQ0LTPSES-LEYHDCFS-GFQCARLD~VPMDYHRSD
          	     ..            .   :  : *    :.:. *:    :****: :***: ...

MG05855.1 	S-------DRAVIAVIRLNATSHTD---YRGPVFFNPG0GPGGSGVWSMLDHGHLLQKVI
NCU00825.1	ATNSSASERRVNLAIVRLRAAVPSNSPDYRGPVVFNPG~GPGGSGIWSLRDHGRNLQTIV
FG06725.1 	DR------KRVVLGVIKLPAKSKNN---KVSPLFINPG0GPGGSGITFVKREGHILQAIV
AN8498.1  	GQG-----ARMAIAVARLPAKVPVTDPRYGGAILINPG1GPGGSGVAQALISGRNLQAIA
          	  .      *  :.: :* *     ..   ..:.:*** ******:      *: ** : 

MG05855.1 	GDN------------H0DIITFDPRGV1GASTPLINCWPTDQQRTFWSLGEV--GVSDSH
NCU00825.1	GTN------------H~DIVSWDIRGV~GASTPRIDCWASTQDRVYWDLQDP--GVVDAH
FG06725.1 	GDN------------H0DVISFDPRGV1GVSTPRMECWGSSQRRRLWSLQET--PVADEH
AN8498.1  	DATVVPTDTNHSNLYF~DIIGFDPRGV~NNTTPGFSCFPNLFSQKNWELQAEADGMLGSS
          	. .  .:.:. :.  . *:: :* *** . :** :.*: .   :  *.*   :. : .  

MG05855.1 	PDVIYDEYYRAYATSQACEANMAK-----SKILEHISTASHARDMLEILHQT--------
NCU00825.1	EGTVFEAFARAAAYSQVCERNLEE-----SGILEHSSTAYHARDMLAILEAM--------
FG06725.1 	PGLIYDAYARATAYSGACESAMED-----MGLMQFIGTASIARDMLEILDKT--------
AN8498.1  	ADSFMRNWQRAIALNPACSQAILTPSVDGDALGKHTNTPPVARDMLEIIERHGEWREKQG
          	 . .   : ** * . .*.  :  .: ..  : :. .*.  ***** *:.  ..  ....

MG05855.1 	---------------------------GEDKLKYWGFSYGTILGGTFAAMYPDKVERLVS
NCU00825.1	---------------------------GEEKLKYWGFSYGTVLGGTFAAMYPDRVERMVN
FG06725.1 	---------------------------GNEKLRYWGFSYGTILGGVFAGLYPERVERLVS
AN8498.1  	FNAQRQFDQMHGYDPQQHILERIKWHRGREKLLYWGRSYGTVLGATFAALFPDRIERMLL
          	 .:. . ..  . ....   .  .   *.:** *** ****:**..**.::*:::**:: 

MG05855.1 	DG1NVDLREWYTHVHTNFPRDADKVMQAFYDLCATAGPSKCAFHSPSPAEIEQRLHGLLD
NCU00825.1	DG1NVDYEDWYNGTYINFLHDTDKVMQAFFTFCHSAGPDRCAFYDSSPNAIKSRLDTLLT
FG06725.1 	DG1NVDYHDWFHLDHGNFVTDTDKIFDAFDTACHKVGPLKCAFHAESPQAIEKRRGDLLA
AN8498.1  	DG~VVDADKYYTGIGPEPIGDADAIFDKFSIYCNEVGTKCPLYAAGGPAAIKASYEKLEG
          	**  **  .::     :   *:* ::: *   *  .*.    :   .*  *:     *  

MG05855.1 	RLRKNPIIIP---------------AKTDQAADGPEIPQIVDYSKLRRYIASALYSPLYK
NCU00825.1	KLRTHPIVIPQGQLPSPSGTPSNPSSPSLASTKPYHVPQLVTYSHIRRMLSTALYQPIFR
FG06725.1 	HLKKSPVLLP---------------AWSQDS--GPELPFLISYSNLQRLIQTCIYAPIVK
AN8498.1  	SLYSSSLPVR---------------------SSETRGPEVVTWSDLKIVQRISVYQPLAG
          	 * . .: :                      :.  . * :: :*.::     .:* *:  

MG05855.1 	FPDFARVLAALEKGDGQPYYKATSAGVPP--LSSFCQATPVEPGAP-----LAAVTESTA
NCU00825.1	FPHIARVLSSLEAGDALPYHKYTLSDYPPPAPSSPTTCEAGDQDDTPPLTFIPKEEAGNP
FG06725.1 	FPQLARVFAALEKGDGVPFYEMVLEMSDN--KAQGGLCELGDTPAT-----TPFETPMEL
AN8498.1  	FTYLAGIVSDLLKGDGSKLADFKHSRRSP--TCPTEECLIAGPWSS----ECAAPGQNEV
          	*. :* :.: *  **.    .           .    .       .      .       

MG05855.1 	DAFPAISCSDSEEWTGTAE-DFVKVGDEIRNLSWAVGAVDTTLRAQCVGRKVRPKFRFAG
NCU00825.1	DVFPAVMCSDALPFTSTPH-AFALYAQKLLNISQAAGAVQASVRLLCAGRTVRPRWEPFN
FG06725.1 	DAFPAIMCSDSKAANESPE-EIAEYFDRITNKSRWAGAANANFRVSCIGRKTRPKWEFTD
AN8498.1  	YSTSAILCSDAEYMQSTDEQDFKRHWRNLQELSSAIGDYWAHTRLNCAGWSVKPKWKLPG
          	   .*: ***:     : .. :     .: : *   *   :  *  * * ..:*::.  .

MG05855.1 	1-~PFEAK--------------THHPILFIANMADNITPLVS~ARNNSQGFEGSVILVQK
NCU00825.1	~S~SLLFKD-----------VQTAHPVLFVSNEFDNVTPLIS~ARKNAKGFKGARVVVQK
FG06725.1 	~D1DFRGD--------------TAHPILYIGNTADNVTPLQS~AFNNSVKFPSSVVLTQN
AN8498.1  	~R~LPAPYQRSRLTTVVPVTANTSFPILLVNNILDPVTPLAS2AKKMSHAFPGSVLLQQD
          	        . :  ::  . : .* .*:* : *  * :*** * * : :  * .: :: *.

MG05855.1 	-SYG~HTSLSSPSTCTASYVHQYFQNGTLP---EPNTECDPDYLPFDNLDTPKIKKYANG
NCU00825.1	GSWG~HTSLAAPSVCMAKVVRRYFVSGEMPGEEKDGVECWGDVVPFGEVYEDVEGQRSDK
FG06725.1 	-AYG0HCSLAAPSTCSVRYVREYFQNGTLP---DKGTVCDSD---FDLFELPVLGEEIMG
AN8498.1  	-SEG0HCTLAAPSTCVSQAIRKYFQTGELP---MPGTVCDADLKPMLGAPKKPQEGKTPD
          	 : * * :*::**.*    ::.** .* :*     .. *  *  .:              

MG05855.1 	GIETKDYDLSDSIKQLCGRAGARLG--
NCU00825.1	DGDENEELRGALWRLLTTRRRRGFGGL
FG06725.1 	LDELSSIALKLSREVEIPRTF------
AN8498.1  	DIALQEAVLDEAQRFRIPLPL------
          	    ..       .
```
